# Supplementary material for: Surface Microbiome Profiling of Dental Elevators Using Third‐Generation Sequencing: Implications for Infection Control in Dental Practice
Source: Microbiologyopen. 2025 Dec 8;14(6):e70178. doi: 10.1002/mbo3.70178 (PMC12685768; doi:10.1002/mbo3.70178)
Supplement: Supplementary file 1 — customer_backup. [file MBO3-14-e70178-s001.zip › customer_backup/customer_backup/taxa_summary/krona/groups/treat/Group.Krona.html]

Javascript must be enabled to view this page.

members
magnitude
magnitudeUnassigned

M01.krona
M02.krona
M04.krona
M05.krona
M06.krona
M07.krona
M08.krona
M09.krona
M10.krona
M12.krona
M13.krona
M14.krona
M15.krona
M16.krona
M17.krona

554814962464805573945319747756483506072357454575246751659664551406100355010

554774962364793573945319047754483306042157454575166751659663550136100354999

21365672148031632151571081

000000031018052600

000000031018052200

00000002006021400

00000002006021400

00000002006021400

0000000000605500

0000000000605500

0000000000605500

00000001106025300

00000001106025300

00000001106025300

000000000000400

000000000000400

000000000000400

000000000000400

213656721480006335145081

213656721480006335145081

213656721480006335145081

213656721480006335145081

5419000130001000000

212500100070270027

276130020007001016

0000000000304400

9410004000700000

492260013000110230022

28300060001300002

630104000000000

387131150008010014

00000001001015900

00000001001015900

00000001001015900

00000001001015900

000000000000600

000000000000600

000000000000800

000000000000800

0000000000002400

0000000000002400

0000000000001200

0000000000001200

00000001001010900

00000001001010900

191816083323034255435611442290140

18027021516342136304712219013

300000000100000

300000000100000

300000000100000

300000000100000

12014000112305701

12014000112305701

200200018002400

200200018002400

001100002202101

001100001201100

000000001001001

1000000000000000

1000000000000000

000100001101000

000100001101000

000000001000200

000000001000200

1787701111634202430077212012

00130111104031100

00130111104031100

00130111104031100

010100017101300

010100006001100

010100006001100

000000011100200

000000011100200

03800029120110200

030004120100000

030004120100000

03500025000010200

03500025000010200

0301021140050180012

0301021140050180012

0301021140050180012

2603070272131000

100200011012400

100200011012400

010003000000100

010003000000100

050104000000000

050104000000000

100000016201500

100000012001000

000000004200500

1785220312410029200600

1784170312210029200600

1784170312210029200600

150002000000000

150002000000000

116906181240041852432100127

0040000001703000125

004000000000000

004000000000000

004000000000000

0000000001703000125

0000000001703000125

0000000001703000125

116902181240041835421002

5151111290001000100

5151111290001000100

5151111290001000100

22160301010410100

22160301010410100

22160301010410100

540207000200201

540207000200201

540207000200201

16100603000510200

16100603000510200

16100603000510200

684516010003181422401

684516010003181422401

684516010003181422401

3051963497867145571321017221243544021596162

267165315431401635796573232614402

267165315431401635796573232614402

01000000000311014220

01000000000311014220

01000000000311014220

267164315431401635796571226182

1661152291119129540381500

1641132291119129540381500

220000000000000

813429202144267418349182

2632382142672403111

51301120120021431801

0050000000000170

410001000000000

20150507000710200

20150507000710200

0022100030103711558043

0000000100107200

0000000100107200

0000000100107200

0000000100107200

002000029103311417043

0000000220000000

0000000220000000

0000000220000000

0000000000108900

0000000000108900

0000000000108900

0000000000103000

000000000000300

000000000000300

0000000000001100

0000000000001100

0000000000101600

0000000000101600

00200007103111298043

0000000100305300

0000000000204600

000000010010700

0000000000104000

0000000000104000

00000000003020101

00000000003018101

0000000000002000

0000000500160605033

0000000100207700

00000002002012008

000000020030140023

00000000009026802

00200001002115105

00200001002115105

00000000106024804

0000000000108704

00000000105016100

00021000000306900

00021000000306900

00021000000306900

00021000000305700

0000000000001200

000000150011046502

000000150011046502

000000150011046502

00000005009041702

00000005009041702

0000001000204800

0000001000204800

02419036404053213145110182515666

02419036404053213145110182515666

0116000022111134256306

000000000000400

000000000000400

0000000300105300

0000000300105300

001000010011100

001000010011100

000000000010300

000000000010300

00000006104408200

00000006104408200

0001000001111700

0001000001111700

010590001200105040306

010590001200105039306

0000000000001000

0000000200104000

0000000200102600

0000000200102600

0000000000001400

0000000000001400

00000002000017000

00000002000017000

00000002000017000

023084364040272023168105215660

000000015003101052060

0000000100101704

00000001300240881042

0000000000003400

000000010060120014

0230843640401220206801560

0230843640401220206801560

38700050904121527020

26000000901121521020

26000000801121486020

0000000200005900

0000000200005900

26000000601121427020

26000000601121427020

000000010000400

000000010000400

000000010000400

0000000000001900

0000000000001900

0000000000001900

0000000000001200

0000000000001200

0000000000001200

000000000000300

000000000000300

000000000000300

000000000000300

1270005000300000

1270005000300000

1270005000300000

1270005000300000

000000000000300

000000000000300

000000000000300

000000000000300

0000000000001029

0000000000001029

0000000000001029

0000000000001029

0000000000001029

0000000100201100

0000000100201100

0000000100201100

0000000100201100

0000000100201100

0000000100201100

3102010002000010

3102010002000010

310201000100000

310201000100000

310201000100000

310201000100000

000000000000006

000000000000006

000000000000006

000000000000006

000000000100004

000000000100004

000000000100004

000000000100004

30310000082833021

30310000082833021

30310000082833021

0000000000006017

0000000000006017

0000000000006017

000000000420600

000000000420600

000000000420600

3031000001081104

3031000001081104

3031000001081104

0000000000001000

0000000000001000

0000000000001000

000000000300000

000000000300000

000000000300000

4232254953001721252255185324740722

4451632045107121020350254

4451632045107121020350254

4451632045107121020350254

02100010007001017

02100010007001017

2208610171077205820121

2208610171077205820121

164700900037013010103

0080000001000010

16390090003601301093

11720101800050151013

1890090004015005

0911109000100108

3124100020141726014

3124100020141726014

3124100020141726014

3124100020141726014

3124100020141726014

4161793082701270231541673224430454

4161793082701270231541673224430454

4161793082701270231541673224430454

41117010130008907510100

28107010130008707510100

1310000000200000

366156217260103023031672414390341

00000001300204700

1012000000800002

130541002103907016651893890190

000001000800001

235102105506303013405230148

16140070007062013

16140070007062013

8670040015400100

847002000300000

0200020015100100

1025460443391361043792013893125554012626

25296111708338173120163850173

25296111708338173120163850173

0000000000001100

0000000000001100

0000000000001100

25296111708338173120163740173

25296111708338173120163740173

25296111708338173120163740173

0200000520140136025

0200000520130127025

0200000001208300

0200000001208300

0200000001208300

000000000000500

000000000000500

000000000000500

000000052001039025

000000052001039025

000000052001039025

000000000010900

000000000010900

000000000010900

000000000000400

000000000010500

77336233232135275101982682519231712428

53621913815112400221433751500133

44820813012111200211293751490133

4261840100890008010301

11339010170002600100

237132080630004610200

76130109000800001

222413021230021492751460132

1540003000300100

616842118009252640048

144600200021011145084

0000000012000000

8811830120011400100

8811830120011400100

8710830110001400100

110001001000000

12120000000500101

0100000000000100

0100000000000100

0100000000000100

1220000000500001

1220000000200001

1220000000200001

000000000300000

000000000300000

19612412316123935101761122210416112259

10368013000260430057

206003000500006

206003000500006

0015000000100006

0015000000100006

8347010000200430045

002501000040310015

8322000000160120030

000010053002010100

000000000000500

000000000000500

00001005200208500

00001005200208500

0000000100001100

0000000100001100

2046000010301654440192

0000000100002007

000000000000107

0000000100001900

20460000003005400185

000000000500000

20460000002505400185

00000000001602400

00000000001602400

1841219161390061765647161210

1841219161390061765647161210

622114070101311400

1221008121383051764336121210

29771101000080135035

29771101000080135035

0014000000300004

0014000000300004

000000000000500

000000000000500

310101000100000

310101000100000

266570090004010031

713002000100000

195540070003010031

0000000000012000

0000000000012000

000000000010200

000000000010200

000000000010200

000000000010200

000000000010200

1803896801474514447149012623246627609434833046137672011317878244629294

65324375908192512853433855286197146836591228512782420319

0004300040000100

0004300040000100

0004300040000100

0004300040000100

104763211507643234750575150593

61321020003601270035

61321020003601270035

61321020003601270035

97742441107443233480349150456

1201020103033006

1201020100025006

000000000308000

0412101000270370045

040101000260280024

00120000001090021

001000100010040030

001000100010040030

9668222907043133080275150375

130006000100101

9565222906443133060275140364

0000000001000010

11453000009109900102

11453000009109900102

11453000009109900102

4901501121819454722320209100173

4901501121819454722320209100173

24083881215407115401599098

010000010100305

24082881215406115301596093

246582460375400740420075

246582460375400740420075

490003001408100

490003001408100

734171201871002311711021

006000000000000

006000000000000

006000000000000

73411120187100231511021

2210104000400000

2210104000400000

010002000000000

010002000000000

220100000000000

220100000000000

49371100181100191511021

49371100181100191511021

0000000000012000

0000000000012000

0000000000012000

13560644144200550931040

13560644144200550931040

13164001100240920029

503000000200000

710001000100000

1061000100210920029

122590414310031011011

400001000300000

500000000400000

1600016000000100

112530412610024010011

000000010202413018

000000010102413013

000000010102413013

000000010102413013

000000000100005

000000000100005

000000000100005

685451489692833178346443925968036907565

685451489692833178346443925968036907565

008100100201000

008100100201000

18302665015126232126690253

1121103110240615028

3100000009016009

1358200000890880082

1187108006801406

127180104010102110000128

000000000000300

000000000000300

2069200000880560094

2069200000880560094

01900000002026000

01900000002026000

13133012001119020000197

13133012001119020000197

0050000006019002

0050000006019002

005000000000000

005000000000000

9300000000000300

9300000000000300

2950222036000191122030

200002000500000

410100000000000

920003000500000

530610190004061010

1416001000010002

4100020001000018

4120009000406100

000100000001100

000100000001100

1069975520170031258618862201019

1240003000500000

4958461170540383721532130659

26261211190016507940124

1911173204003144027550236

430000000000000

030000000000000

400000000000000

360212354957618857825329025551702505779

040002000000000

000001000008002

017000201120000000

008200000067013910116

510000000300004

021002000900100

54900000301990001

000000008000000

000000000100102

13112001100140230021

040007000106100

0160000007060021

2282973310013298046950474

57207110160006201420079

0070000001000021

000000007000000

627103102206031000

515905210302192701364201546

21437661102421416850988901295

1100000001000015

962111691511813010181551523101851

0000000000000010

30000350160200000

1927106011270193020

558202429000151045200288

2300000020110002

120001000400005

040002000000006

000000024200000

007000000200100

723584402000295116640191

461012080001100000

2600020002011207

000000000006000

241983201000282114920184

60170000102203416016

609000000210340016

609000000210340016

609000000210340016

0080000101001600

008000000100000

008000000100000

0000000100001600

0000000100001600

44591808204751956136564512813177461062602517103246

158611861205403021212101560222

0000000001600000

0000000001600000

682815403400044119404

101500300036019400

672804031000810004

201100000080150011

201100000080150011

0000000200006200

0000000000004000

0000000200002200

6027104010170140020

3027102010130140019

300002000400001

82331337016000127018000187

28829205000420230054

225000500090190020

322010450600076013800113

00900000014000027

00900000014000027

00900000014000027

6952133411583440335134340323

12317006000210710048

420001000000000

8117005000210710048

38416451383440211013540151

7270302700047054040

164140153360820260020

006000000708000

150105000300005

410100080501000

0044000000620940086

120001000001000

920000000500000

1985236014000103113700124

1985236014000103113700124

1530000000100000

1530000000100000

1530000000100000

100004000400000

100004000400000

100004000400000

1321102000400200

1321102000400200

1321102000400200

24503011000500007

24503011000500007

24503011000500007

007000000600100

007000000600100

007000000600100

113829749528440210304399744676174981070991

0030000100001000

0030000100001000

00360000005015009

00360000005015009

010000030010100

000000030000000

010000000010100

3681141788391331939283569041

3371061748351331939276568041

3180404000700100

201341030110185019300185

001341000110179019300185

200003000600000

79312841925930521269333940259

79312841925930521269333940259

0000000200103000

0000000100002100

000000010010900

0000000000004200

0000000000004200

103010210137000014

103010210137000014

5359824824871376921071319480372

2144201000200110017

37576391015301211412823097

31512310753757082207350197

8713020110002912002

3310001001800000

004000000100005

7238000000500260054

00600000013000018

00600000013000018

1437855965704242313674513090

346034300300000

3023000000390101013

000000000607000

0114000000270250040

361301010000420000

27100007010500000

3230102000700000

230000000400000

1000000000100000

1532109101501732070014

33100101211510000

1291323112393224122023

1000000002200003

1000000002200003

13496888044000135023030103

10350206000300000

10350206000300000

1310030004015108

1310030004015108

533353402000010201691070

533353402000010201691070

7021020100001200000

7021020100001200000

0434005000140461025

0434005000140461025

003000000000000

003000000000000

003000000000000

2629120140001100207

2629120140001100207

2629120140001100207

000000000200008

000000000200008

000000000200008

1250005000900010

1250005000900010

1250005000900010

237798374570701214108830109

391611115070906005

123100070101000

530000000200000

33118015000605005

0000000000407106

0000000000305704

0000000000101402

30900000033071906

30900000033071906

19563636352000790313092

4180109000500000

1374918423500037024308

203401100013010046

1561110700024060038

1946640505101416745890171

1425722304201315535878052

14257213042031540571030

001000010013177022

52918209010121012119

5291820901012000119

0000000000101200

15264472305533193106753528198107277

35121020600018010116

1253003000501000

237720300013000116

1584535204710036001013

1584535204710036001013

44813894252932321141613533

44813894252932321141613533

136120160053900000

115110150053400000

210101000500000

4130010000200000

4130010000200000

410000010303402014

410000010303402014

9624000000180200040

9624000000180200040

020001000500000

020001000500000

855224662401560002692711460171

5814211016000540170047

79721045230140000215279760124

13856040350003201101

13856040350003201101

120002000000000

13754040330003201101

77461077197850128555491577519565811100

49179312921510013771439260352

49179312921510013771439260352

0180000001500000

0180000001500000

0120000005012000

0120000005012000

321318209000330140010

001800100027014009

200001000100000

30130207000500001

000000000000004

000000000000004

57631536424123916375700430290536

471432201100024030003

169441540340004600200

7613002000700002

010000000000003

1249013960761524178092180130

2128001000370250061

214312010000103018400106

00560000006202900103

00900001090180037

336001500011015004

1901311810096103830329074

282300400010050013

000000000000005

000000000000005

3027000000210120026

3027000000210120026

203314611908152148341024

12930160813800005

302600002248340019

41400011000900000

1109002000200100

300001000000000

300001000000000

000000000200001

000000000200001

40120108000300000

40120108000300000

51562655474125442653021521142

51562655474125442653021521142

0402100000010015

0402100000010015

0000000000000015

0000000000000015

040210000001000

040210000001000

9461213581729111024212168514307

2580104000700100

960002000300000

960002000300000

1620102000400100

1620102000400100

6953213571689111023512168414307

461595211400012008000161

3181010008060014

1165101000201000

7350016000610550092

25532006000490180055

77331105000310351046

0470000004011004

73261105000270241042

106271030002003082019

11010000050308100

3010000002001015

51260000001300004

140003000000000

2111320110001402011422

2111320110001402011422

414454103591110501510059

114041034911101118000

3045001000490330054

000000000000005

1137000000130151017

1137000000130151017

1137000000130151017

1137000000130151017

102610101126717168027

102610101126717168027

1022000000200171103

000000000000400

000000000000600

102200000020017103

004101011670154024

0001000110204100

00400100062030022

0000000000308302

000000000000300

000000000000300

1000000000300100

1000000000300100

1000000000300100

1000000000300100

050000000000000

050000000000000

050000000000000

050000000000000

0000000003011002

0000000003011002

0000000003011002

0000000003011002

201301040007406310135

201301040007406310135

201301040007406310135

171301040007106300133

300000000300102

200000000300000

200000000300000

200000000300000

200000000300000

0000000000101700

000000000000600

000000000000600

000000000000600

0000000000101100

0000000000101100

0000000000101100

0001600000300001

0001600000000000

0001600000000000

0001600000000000

000000000300001

000000000300001

000000000300001

255136598565923911181341542212362406498

255136598565923911181341542212362406498

0000011001000056

0000011001000056

17337107000391171041

1710003000400000

0237104000351171041

901259135359222538112022014606218

901259135359222538112022014606218

19918108079112605312022

20141027801110179010

70000200040191011

820000000300000

120007000500101

044002000000000

0000010101017100

010000100200000

1000066000000000

020000000201000

020000000201000

384011028000270663012

3191001100010048105

27190101400017018207

820003000000000

410000000000000

410000000000000

70462900430115808520145

108220017000170330094

52220001800011018100

8167008011300341051

000000000000004

000000000000004

1750007000100000

1750007000100000

006000000000008

006000000000008

006000000000008

006000000000008

371220961130402970323870375

9310102000140270040

9310102000140270040

9310102000140270040

0000000100007500

0000000100007500

0000000100007500

6200030001200007

6200030001200007

6200030001200007

8222001000500004

8222001000500004

8222001000500004

20150412030168017210241

20150412030168017210241

20150412030168017210241

210001000200000

210001000200000

210001000200000

200002000100000

200002000100000

200002000100000

114000000800000

114000000800000

114000000800000

732310200087012411083

732310200087012411083

732310200087012411083

15033810288207655311087163104624101262

15033810288207655311087163104624101262

0000000000001200

0000000000001200

009101000120330012

009101000120330012

000000000010400

000000000010400

000000000000800

000000000000800

005100002044185190111

00510000204416980111

00000000000161100

009000000000000

009000000000000

0000000100001900

000000000000300

000000000000600

0000000100001000

0000000000001400

0000000000001400

000100000000300

000100000000300

0000022010010500

0000022010010500

001000201511100

001000201511100

441413109100440402058

311313108000440402058

1310001100000000

0000000000001200

0000000000001200

010000000000005

010000000000005

42933430514015222033023

02441073001801000

00110000003000021

327182030000700202

00000121000301000

100002900151101100

29271867160203214727598673301028

1235200024000180253020

9693135038000278137280463

816753411014121443158470220545

0001800000000000

0001800000000000

210001000100000

210001000100000

7122452015011316017106025

2010002000400000

5121212013011312017106020

0024000000000005

115065305566413522136272802081223234151183631310878281660024228975

0000000000001100

0000000000001100

0000000000001100

000000000000800

000000000000300

001600000012125608

001600000012125608

006000000000000

006000000000000

001000000012125608

00100000004125600

000000000800008

4711030110101501207

4311030110101401200

4311030110101401200

4311030110101401200

400000000100007

400000000100007

400000000100007

859133083289108009902345101914071231811204120725212906320135204

200021026112311041183567

100000000010801

100000000010801

190021026112311040103566

190021026112311040103566

10290000290340780024

0000000290000000

0000000290000000

000000000700000

000000000700000

1029000000270780024

1029000000270780024

110951604005038113023

1100000000100009

000000000100009

1100000000000000

009000000000000

009000000000000

00000000040380014

00000000040380014

0005100000000000

0005100000000000

00006040000011300

00006040000011300

14147404000200000

14147404000200000

14147404000200000

642701025000900400

642701025000900400

642701025000900400

102524980250106401625037

99524960250106401625037

99524960250106401625037

300200000000000

300200000000000

24021773168825384089944077848558665648652549495472702

640103000100100

640103000100100

710205000100000

710205000100000

216115814942397407839320783350052244863131548450823

216115814942397407839320783350052244863131548450823

188844641260908711535351335940699

132291032021130228053

01081120312144062401

9033428028855276016544045

8519405502712114580242180600

401037301215704589518991471180

100000000200000

0049000001640460068

10150000008000030

1000000005011009

0028002000290800034

14334097115023430146260691

1152001000340340054

034261011000480422081

221600300011000013

09413040111120563625

4700010001025015

00100000010010011

4640107010410750045

20700000047030056

0011000000200000

1011350013000510643058

1023915860343001261146230103

410001000100000

410001000100000

281361060001711205

281361060001711205

424914072007501001059

510102000000000

151803031006901000059

22211002100600100

20022003100150150017

20022003100150150017

5200010001001300

5200010001001300

319391016000170307022

000000000000700

319391016000170300022

320002000703009

320002000703009

320002000703009

365861122183454260709276798496

1000010001023000

1000010001023000

310061500200000

310061500200000

30000001010009848

30000001010009848

118370010003530536070

0000000001017003

100001000200100

01837009003500365067

283861220224205213201018

28380001400110001011

0060004200300007

000122080051020000

011801207150900000

011801207150900000

771445486126821511019001127661

135401971611970001450

135401971611970001450

0000000000000026

0000000000000026

0310060309200006835

011000000650000530

0200060302700001535

7678148611124083000101630

0000000022000000

767804861112401230010490

0010000068500001140

1356472601501011708810134

1356472601501011708810134

1356472601501011708810134

5485105612083178532686469592249939557109206139373682006

0005800000000000

0005800000000000

433100100024012006

300000000100000

032001000400000

102900000019012006

20725102000110100017

20725102000110100017

139181310016066605231132030

139181310016066605231132030

0084102000440300016

0084102000440300016

13441118403502015108910165

279810200011408100148

4217020140001000100

901720101902027080017

3834540110320516041642110808

3320870016000122015070111

51445311016051482149240697

1100000000408000

1100000000408000

3843159842415820514218612731061386104142

3320000000000100

3512959842415820514218612731061385104142

03700000000000220

03700000000000220

17313210711084010010102

110000000100000

16303210711083010010102

125734274188426213316146811927513209

04391030004109900112

10344264158426213312046820275096

11100000000000131

27510050025001019

27510050025001019

173112242801939890212115240109

0000780700000000

17311223501239890212115240109

4677840221255920844016085434521653658802256229383

4677840221255920844016085434521653658802256229383

510000010100000

510000010100000

13979340516094181554910222

8422030200002400200

8422030200002400200

555731049609418131498222

44000000011000170

11573104960941803049852

246913471351403612595125400147

21969305034100580292038

14035181015000340291029

12427171011000240280028

16810040001001101

68321230181002000109

68321230181002000109

1120101000400000

1120101000400000

27224211040332592225380109

27224211040332572224940105

260000009102190836023

12242110312338221658082

000000000000700

000000000000700

0000000000203704

0000000000203704

365300038000900301

365300038000900301

365300038000900301

365300038000900301

00000001201146211082034

000000060113521635020

000000060113521635020

000000060113521635020

0000000600110447014

0000000600110447014

0000000600110447014

04800199013582600001680

04800199013582600001680

04800199013582600001680

0000130081800000550

048006905402600001130

010101000300000

010101000300000

010101000300000

010101000300000

10001003124015600

10001002124014300

10001002124014300

1000100102108600

0000000110304100

0000000000001600

0000000100001300

0000000100001300

0000000100001300

0000000000001100

0000000000001100

000000000000800

000000000000800

000000000000300

000000000000300

11364644423831533443807321092368945735336

0113001000200001

0113001000200001

0113001000200001

1520182751430901827255219871719

112001901702018

111001801602015

001000100100003

000000000106006

000000000106006

000000000000300

000000000000300

100119143069800510000

100119143069800510000

13191615602012714221804165

00000000002130000

091000000200100

1061801000100000

12109138019127139503165

000000010000200

000000010000200

000000010000200

0000000000001300

000000000000700

000000000000700

000000000000600

000000000000600

25000000000002000

25000000000002000

0000000000002000

2500000000000000

0000018000000000

0000018000000000

0000018000000000

0130000000010900

0130000000010900

0130000000010900

0001800100000000

0001800100000000

0001800100000000

737251102443154344120511053918298

737251102443154344120511053918298

20100006502000000

0000024000001000

6761999231714377911948749718295

4151112150008333203

0238390103301528396013

00000002000103800

0000000100002700

0000000190000100

0000000000101000

00000000000285702

00000000000283002

0000000000002700

02202901090040271011

000290004004013607

02200000200008104

0000010300005400

0101000030100000

0101000030100000

0080000100003000

0080000100003000

000000000010105

000000000010105

000000000010105

000000000500000

000000000500000

000000000500000

000000000500000

0000000000001700

0000000000001700

0000000000001700

0000000000001700

58240101700013102800

58240101700013101700

000000000000400

000000000000400

0000000000101300

0000000000101300

5824010170001300000

5824010170001300000

0000000000001100

0000000000001100

0000000000001100

0000000000104800

0000000000104800

0000000000104800

0000000000104800

00126700061010121308

00000004005010900

00000004005010900

00000004005010900

0012670002105110408

000000000011500

000000000011500

0000000000203608

0000000000203608

001267000210206300

0000000100101000

001267000110105300

00600006001015200

00600006001015200

00600006001015200

00600006001015200

206415903342152114159269218811717163034473157020633

361214490102000

251102240100000

251102240100000

110112250002000

110112250002000

100000221002101

100000221002101

100000221002101

533200312010399503

00000000000098800

00000000000087600

00000000000011200

5332003120103703

5332003120103703

000000000010202

000000000010202

000000000010202

193999478402947561917968112510455224223

1826959112750365579803373532919603

1826959112750365579803373532919603

1255424314708461022010

1255424314708461022010

812261442736170951315166210

812261442736170951315166210

208567115200061300000

208567115200061300000

3212200592228313200

3212200592228313200

2212000591228100000

102000100213200

10137321327231051011737710000

10137321327231051011737710000

10137321327231051011737710000

50000000011400000

50000000011400000

50000000011400000

63210214362923200

63210214362923200

63210214362923200

116116147499258131671023553442044

100000040036020592

100000040036020592

000000000003000

000000000003000

0011524300199013811

0011524300199013811

00034000085000000

00034000085000000

0320105831026024500101790

0200000000000000

0120748020800001260

000023024070000260

00098400002300010270

01291538163028613410011151

01091534263028612610011151

020039000080000000

000000010000300

000000010000300

000000010000300

000000010000300

0000000700006100

0000000000003000

0000000000003000

0000000000003000

0000000700003100

0000000700003100

0000000700003100

516501220021090210016

516501220021090210016

516501220021090210016

21100121002600000

000000000300000

020001000200000

3000000007500000

0000000009021006

000000000800009

030000000000000

005000000600001

0000000000002400

0000000000002400

0000000000002400

0000000000002400

0000000000002000

0000000000001100

0000000000001100

0000000000001100

000000000000900

000000000000900

000000000000900

19443180045645186183193070022438003000

3714385812020544049010708

2164200000193016600164

2164200000193016600164

433410000051020038

430000000100000

003410000050020038

34287400010294032210506

34287400010294032210506

1150112000200000

1150112000200000

1710000010400000

300000000100000

1410000010300000

15729141537543186182992526017537902292

17038381861824085502774018

17038381861824085502774018

4140010106000016

4100010002000016

004000010100000

000000000300000

1520000000190341038

010000000140341038

1510000000500000

7120384023002300140024

10100108001000000

102100000013040014

60105201500113010000

00121000004000010

506137025211047161601678402196

400100000000000

300102010000000

00387310000477056700620

003000000107000

005000000604006

3000000000014000

005021010000474053610759

004110000013109300112

004610200033010043

63010200080160025

32110020102900108

1229202007420262037

10356501020415041400586

0001000003301000

000000000030400

000000000030400

000000000030400

000100000300600

000100000300600

000100000300600

0000000200002600

0000000200002600

0000000200002600

0000000200002600

0000000000204900

0000000000203900

0000000000203900

0000000000203900

0000000000001000

0000000000001000

0000000000001000

0000000000001100

0000000000001100

0000000000001100

0000000000001100

1505617292113191472022648068

1505617292113191472022648068

13956112021000400210018

111110020007000012

9343020100001401001

3512000900019020005

000000010000200

000000010000200

000000000400000

000000000400000

000000050000000

000000050000000

006000000000009

006000000000009

1100090131310201646031

110003033006014404

0000000100005800

00006010910141444027

0000000003000010

0000000003000010

000020100000600

000020100000600

000020100000600

000020100000600

001000000400000

001000000400000

001000000400000

001000000400000

0086110035059756198083

0060000000101500

0060000000101500

0060000000101500

0000000100203200

0000000100203200

0000000100203200

0080110034059456151083

0080110034059456151083

0080110034059456151083

000000015011091013

000000015011091013

00000004011059013

000000010000700

00000003011052013

00000001100003200

00000001100003200

100000050601314017

100000050601314017

100000050601314017

10000005060138017

000000000000600

5332566131851810331430234726400541

125154436651000100295143154050399

125154436651000100295143154050399

0000010001018000

0000010001018000

0000010001018000

12515443665990100294142974050399

621270121000130282026

621270121000130282026

4800090001201000

4800090001201000

1141004000210250055

1141004000210250055

19530103000410590069

19530103000410590069

5319331480902914824000116

080001000101361018

531133147090191346399098

21082108000340180013

21082108000340180013

005000000000000

005000000000000

4090218304601014408430120

388916820440001010243016

0039101010330580078

211100100010020026

40810217712081023113591572350142

000000000000705

000000000000500

000000000000500

000000000000500

000000000000205

000000000000205

000000000000205

1190000003000020

0090000002000011

0090000002000011

0090000002000011

000000000000009

000000000000009

000000000000009

110000000100000

110000000100000

110000000100000

173417570370231787129186055

6520445016001270450024

9500090001020000

450008000100000

5000010000020000

900000000000008

900000000000005

000000000000003

820101000301000

110001000101000

710100000200000

391344406001230240016

034420200170230011

900002000300000

301002020001301005

010001000200800

010001000200800

010001000200000

000000000000800

0000000000101700

0000000000101700

0000000000101700

901017106023035665161030

1070000002010104

0040000001010001

103000000100103

891010106023033655160026

0000000130069158011

64000000001400000

259101060100110462015

010000000800000

181014101400014019001

640006000200001

640006000200001

1261410800012019000

1261410800012019000

000000000000300

000000000000300

000000000000300

000000000000300

193607650440004522711033

193607650440004522711033

193607650440004522711033

520000000000000

1910002000300000

9129020170001920100

78287630250002302710033

0000000000001800

0000000000001200

0000000000001200

0000000000001200

000000000000600

000000000000600

000000000000600

4100000000000100

4100000000000100

4100000000000100

4100000000000100

000000000000900

000000000000900

000000000000900

000000000000900

00170000009010029

00170000009010029

0040000000010018

0040000000010018

00130000009000011

00130000009000011

4274730230002601496084

4274730230002601496084

4274730230002601496084

4274730230002601496084

4274730230002601496084

050005000000000

193310800030643032

2180000004032003

11262010000190533049

0000000000000010

0000000000000010

0000000000000010

0000000000000010

0000000000000010

0000000000000010

409341041418316593961089499569950143603298121664105456114442400

000000000010700

000000000010700

000000000010700

000000000010700

000000000010700

408741011418316493961089199569948143603286120763785269114442387

506276103476122811919966126107810801458

1149003000403001

1149003000403001

1149003000403001

49526910057612251119936110203201369

220001000110480010

010000000100480010

210001000100000

31653130630011211700

0111005000300100

1015291021001510300

213912037000401300

63631013000250430029

3320109000600000

103000000110430029

2420002000600000

520002000200000

1155413502700027010106

500000000400000

106541350270002101106

400000000209000

4760109010500000

4760109010500000

100000000200008

100000000200008

447952456059107417029590573

111300014000400001

7101600600025001012

5417004104130211023

1110010700010001013

18000400016062023

14224577017003328025230476

031003000500000

51004803000500100

013001000110160025

72181602114000149019700257

930000000100000

1319600200079011300145

32864118000600690079

01000000050150033

1850104000400000

0010000003015000

0010000003015000

340001000000000

340001000000000

0149103000260211034

0115003000120140012

000000000300000

003410000011071022

395222040009007220128

395222040009007220128

330008000000000

330008000000000

71181062012000102016400185

2084000000350610068

691822201200067010300114

000000000000003

489630110011670154120139

489630110011670154120139

03200000180261255576088

03200000180261255573088

0100000180112506304

00900000014000012

0211000000110550072

0000000000001000

000000000000300

000000000000300

672451017558266917263168812224216694161

040000000000000

040000000000000

040000000000000

46182727447166459252911521469411

000000010010800

000000010010800

000300000000000

000300000000000

000000040000000

000000040000000

000000001000500

000000001000500

2100610300360001102

0000030010001102

00040000210000000

210021000140000000

000000060000000

000000060000000

000000010000402

000000010000402

0000000100002100

0000000100002100

13012000002014000

1000000000014000

1201200000200000

018107444166422400006940

012102120700002270

00005005000000000

0600636225552000004330

000056057000000230

000000006000000

00000000110000110

0000018000000000

12000000100001200

12000000100001200

000000000010700

000000000010700

0000000200004307

0000000200004307

000000000000500

000000000000500

0001000000007000

0001000000007000

00000004008903100

00000004008903100

000000010000700

000000010000700

000500000000000

000500000000000

8001460450000000

8001460450000000

0001400050000000

800060400000000

000000070000008

000000000000008

000000000000008

000000070000000

000000070000000

13231551111655668631920142

000000006000000

000000006000000

0000000000310000

0000000000310000

1323155111153006860920142

1323155111153006860920142

00000001250000000

00000001250000000

335036291352915847510446917681921428413898834768474210723279

00001204100204301

0000000000204301

0000000000204301

0000120410000000

0000120410000000

110005086000000012

110005086000000012

110005086000000012

0000701000000000

0000701000000000

0000701000000000

11149281819575399777622510346440137831663296146

83400015200201203201

83400015200201203201

4438512241027303033306

4438512241027303033306

35612634828712678751512264413441

35612634828712678751512264413441

70673010146453533515411978138610873124529298

70673010146453533515411978138610873124529298

27115218203348940400808415886813413

00000000000121152

0000000000000152

0000000000012100

5100002000000000

5100002000000000

5926013733033000001751160

02601370000000001060

000020000000000

590000000000011400

000031033000000100

0000000000006100

002000101002000

002000101002000

207116166618764008074155672311

300000071200030

70401613074001120806

83440120130501422700

1960302000100200

320101000200000

230303001530032002

0000000000006000

27150515231772941327503

0602904904962400000

0000070220000000

0580311005181078310005203330

0580311005181078310005203330

0580311005181078310005203330

253136136042724040632140

630002000100100

630002000100100

000000000000040

000000000000040

247133136040724039632000

1780001000320000

34220705000201100

196103129034724034421900

00070004102012905

0000000100104804

0000000100104804

0007000310108101

0007000310108101

0000010210000000

0000010210000000

0000010210000000

9897682717791788729452368381664033924446

9897682717791788729452368381664033924446

000007000100000

1004100100001226

60011016000018018

531134108091217522112208

78845081350144282169251784312

0002030135962000181

20000001231000690

060000000000000

100000010100100

0000000000120000

22110121073936792097542718004

0101100014000900

43013412510482110283

0002201107147500160

01300050000101102

000100010000200

1127231602459312033802

51847177976527617359620180143292164165915

955611301710028531301

955611301710028531301

013717336511670258190125275015811

01010716596166389000275011831

0361175506449190125003980

42327855117283238115213141517813

13792114480131503655583

1276022302400031231301

90430602031102233700

97500502241210241166209

200000000000100

94461160120302541900

193883645884808369750824056403075051256951220827

0100000800010660

000000020001000

0100000600000660

503111018021891516030111017

000000000008000

503111018021891516022111017

864893775192878526501212418367821133

000056041000002900

000100037121001100

8648956162878231302418565203

00004440461000031001130

0032030100000000

557712254901073155008111723

557712254901073155008111723

100000120100002622

100000120100002622

0000000000000300

0000000000000300

114925319215012222516108016169313012671

00001802600000030

11204745104340000004601

0120085121060700294120

001301850196000000190

0007960814902426934270

006000000000000

00001770160000000250

00001701200000000

00001201300000000

000040500000000

06900800800000360110

03901711005000020555920

0000600440000001050

000039044000000360

006000000000000

00000000000002590

0170221006010921552013715402280

013602592137782328158003361575173981

00000000000022300

0100052056000002310

041005613490000401450

00002090255000030020

00019408043110006900351

05013208020800026701190

00013300000000050

0000000000000170

000000001000020800

0001300000003000

08003515636640005652540

0250062364000053000

00002502806000000

0190810601640000500

0280019002080000111050

00005760663000000650

0000000000007600

0000000000007600

0000000000007600

0000000000306200

0000000000306200

0000000000306200

121705891631531740071013296541555862

121705891631531740071013296541555862

10000000001201000

018405761053573011016925501

000810000250000210

0604519900113503500227335055631

0210000000000020

06003103200000000

0000000000101100

0000000000101100

0000000000101100

016029223702580300069025912

016029223702580300069025912

016029223702580300069025912

61727116371101111085282726

000000000000006

000000000000006

000000000000006

6826107001110330020

463105000700006

300100000100000

003002000600006

160003000000000

221700200140330014

221700200140330014

006000000000000

006000000000000

0910163011000005226270

00000000000141200

00000000000141200

000000000000600

000000000000600

000000000000800

000000000000800

091016301100000380270

000024022000010000

09101390880000280270

000000010000200

000000010000200

000000010000200

15815521771212801025232764231720463

24261430250031601274031

0010000000000003

0010000000000003

140003000000100

140003000000100

0200020002012006

0200020002012006

2320430200031401153022

0340060001032005

5150301200190483017

18200020024035000

1616145701102061056010

002000000200000

002000000200000

16161210110203105600

16000000000103900

0901060203001300

0712005000000400

0005600000000000

0005600000000000

0000000001000010

0000000001000010

1269102800109124040

020001000000100

020001000000100

0000000002020014

0000000002020014

015910150005101026

0390030003000011

012010120002101015

020006000000100

020006000000100

1700060010200100

170006000200100

0000000010000000

4241000000300000

4241000000300000

4241000000300000

532832321200055112662062

146292140004111230058

146292140004111230058

2773118000800000

2773118000800000

121500000006036204

000000000200004

101500000003036200

200000000100000

22551477052081214363268460320

000000000701402

000000000701402

64853104300060015460153

010001000007204

08000500070200020

180007000200100

217370020000370942088

31300060004001024

0116104000100330017

11215006010400004

1100003010400004

010003000000000

0110000000000000

005000000000000

1590010001101603

009001000600403

150000000501200

407060207126163112300158

0001000001012000

0022000000120230019

0000000001000011

0080000003000012

0027100001320311082

0001010022010012

1000000232031000

0000000000012000

000000000001107

10320003516341200

0051000115017405

103000000000100

102001010208104

000000000000006

000000000000300

000000000000300

000000000000300

000000000000300

00000101043166501

00000101043166501

00000101043166501

000001000400001

000001000400001

00000001003166500

00000001003166500

1101020008031011

1101020008031011

000002000000107

000002000000107

000002000000107

110100000803004

110100000803004

110100000803004

520000010051311401

000000010051311401

00000000002133300

00000000002133300

000000000000700

00000000002132600

0000000100308101

0000000100101400

0000000100101400

0000000000206701

0000000000206701

520000000000000

520000000000000

520000000000000

520000000000000

23268374906302118743442590406

14749316804902114242122570322

50230000102101105

50230000102101105

000000000000705

000000000000705

0012000010010400

000000000010200

0012000010000200

5011000000200000

0011000000000000

500000000200000

00000000002012804

0000000000002800

0000000000002800

0000000000002800

00000000002010004

0000000000001200

0000000000001200

0000000000208804

0000000000208804

000000000000300

000000000000300

000000000000300

000000000000300

000000000000500

000000000000500

000000000000500

000000000000500

142492938049011140021260313

141462778048011139021260307

141462778048011139021260307

141462778048011139021260307

1316001000100006

1316001000100006

1316001000100006

00000000001010400

00000000001010400

00000000001010400

00000000001010400

85195810140004501322084

85194310140003801322084

85194310140003801322084

501014008000190740026

39814004000170500026

11200040002024000

2472410300040541024

2472410300040541024

112500300015041034

711001000500000

41400200010041034

0015000000700000

0015000000700000

0015000000700000

0015000000700000

2500001040050195036

2500001040050195036

2500001040050195036

2500001040050195036

2500001040040182036

2500001040040182036

0000000000101300

0000000000101300

1043879256470314942645056378411648887861109988987291371010880

43048441263145182271322342710637016112528611

43048441263145182271322342710637016112528611

1207031320102001

1207031320102001

1207031320102001

029816730951925325812633421321128858

02700262191781922330079663148

02700228014619220666399141

000000000300000

00000000000156200

000034193200023415207

02711672833023543910404253465710

000027602210000025600

00002296019391800000710

0271102510188211040425320900

00067100600000000

42918435250160168163301102282712412

0006120404000002

0006120404000002

50262115411512087016210

00000301810000000

483000170257401110

250002101225201000

018211131961661014100

000023000201100

000023000201100

6925060140001921200

6925060140001921200

9300841076171300

930080100501300

0000040071170000

0000200800000000

0000200800000000

000001406371017400

000001001311017400

000004056000000

174841223301441044513000

1330204000000000

160810200261430042512900

101030110200100

12746017041154523421200

310101000100000

1234201601410020311200

13000260545211000

000040900004000

000040900004000

15660109613802076070860111

15660109613802076070860111

15660109613802076070860111

15660109613802076070860111

15660109613802076070860111

123842712542181237878726114846146696501972

215275637641235186923697830106

622656364121706923457810103

0000000000105700

0000000000105700

00000002006015003

00000002006015003

00560602304833436067

00560602304833436067

351600021000500001

351600021000500001

0001010201202208

0001010201202208

2510020180804119471012

2510020180804119471012

20000102002065012

20000102002065012

153103400018180024203

153103400018180024203

0003400000000000

15310000018180024203

1946214341250260168121530215

3322292018000250490044

3322292018000250490044

3322292018000250490044

1092210905021041025

1092210905021041025

1092210905021041025

9213223000514100

9213223000514100

9213223000514100

2180304000700100

2180304000700100

2180304000700100

871702012100700000

871702012100700000

871702012100700000

34491104110103015800146

34491104110103015800146

34491104110103015800146

37233205024021251030900465

001100000070120022

001100000070120022

001100000070120022

0060000003000024

0060000003000024

0060000003000024

0230000000400810060

0230000000400810060

0230000000400810060

0060000002070014

0060000002070014

0060000002070014

37192315019021173019500303

37192315019021173019500303

37192315019021173019500303

0236005000260140042

0236005000260140042

0236005000260140042

001000000207002

001000000207002

001000000207002

001000000207002

792315734135426334476582286617901184

26487307010346346730119

2007001002304400

100101002002100

1006900000302300

064330600143142190111

0000000000101400

06433060014304250111

00500001000205008

0000000100000200

0050000000204104

000000000000704

1455615870370011462242470324

1455615870370011462242470324

102103000101300

113132030017015108

1335314340310001382226430316

494190201202156031222016262350357

3420191014000150351027

2161002000100000

1314181012000140351027

44516414818013702801971519750313

2530004000200000

42016114818013302801951519750313

15634125032813029017

2215012021513016017

134191130113001300

15163327352633312461316240384

000000000010800

000000000010800

1450001000500000

1450001000500000

13758327352623312410316160384

13758327352623312410316160384

6048291915122389213213650924131322528032709

433219728351606147013548214651012675621381

4560020003001012

4560020003001012

4560020003001012

164277237846451122406611742200652

130361825134040022204801569130439

5000000100100000

5863004813017805016908930101

0315000000501006

2045910704901047012013

413886302401085021230137

3891835780126250131115750129

78353530230004201090053

014000000100000

014000000100000

242500200016000020

20500000011000020

2220002000500000

255934010071020820914071

30100003000400000

18101010102011000

20782409067010760804071

1333481018000500601092

1012300013000210360062

34910500017090015

0179000000120151015

4725301420000320222030

4725301420000320222030

2686119545111429571332428019525352717

6821492019010551821088

6821492019010551821088

0003100001300705

0003100001300705

259311713898029341332317398443272624

259311713898029341332317398443272624

7013000000000000

7013000000000000

1830104000400000

1830104000400000

171694767778366212794839852411328

11136402635124141274532343120600

11136402635124141274532343120600

1831091310156117501232024

885508250390346010396131980576

452302112000701200

6033074142712480004951642121728

1610005000010000

1610005000010000

24111204000401100

24111204000401100

5632954132512390004910641111728

5632954132512390004910641111728

23243936356325551097224992680142440521056949855266216022

000001010400000

000001010400000

000001010400000

000001010400000

130000000100006

130000000100006

130000000100006

130000000100006

0000000000200000

0000000000200000

0000000000200000

0000000000200000

000000000008000

000000000008000

000000000008000

000000000008000

0010000003010301

0010000003010301

0010000003010301

0010000003010301

151671840460214324612018

151671840460214324612018

5110010003172002

5110010003172002

300000000100002

300000000100002

1592007000400000

662001000200000

930006000200000

1081005010100001

1081005010100001

1110101000300000

1110101000300000

003000000270000

003000000270000

432301010000500000

940005000200000

230001000100000

330001000000000

29130103000200000

291732090111009101

000001000006000

2592205011803101

481003000200000

1843008000300105

1843008000300105

005000000900007

005000000900007

1000000000050000

1000000000050000

1640005000200000

1640005000200000

31012001010150321011

31012001010150321011

0012000010130280011

001000000001005

000000000107000

001100001012020006

060000000000100

060000000000100

340001000204000

340001000204000

15912396617779258201503842167373

16311917298377138402738

1631117298277038402731

000003003000000

0611017268171036802550

000100070000030

12000003301600151

00018000100100007

00018000100100007

135566303300061013204

135566303300061013204

34150008000601100

341800080002902000

191100070001904001

481263010000706103

23424401512211388082061

2011340121204978032055

3114000193201002

1700300121185676022053

100000000003004

100000000003004

23110030174700002

23110030174700002

000000000302000

000000000302000

1098927111667188551174113924401881228

1098927111667188551174113924401881228

033805800459170000090

03380000170000090

0000580045900000000

1094519111681887815711392440188328

001001203330018000

70123717771471518182114317

1611116513108129000

000100200000000

000202000001100

191111711011132252003

010004602200000000

1961319322289111013308

011001901400000000

011001901400000000

1521642468013001652232270150277

1844119204000097112350137

0623002000190380029

0623002000190380029

16309420320007718320108

16309420320007718320108

282006000102300

282006000102300

13915304010200300019

012001000005002

012001000005002

13813303010200250017

13813303010200250017

13141008000200000

13141008000200000

080004000000000

1361004000200000

2070104000300000

2070104000300000

2070104000300000

22120108000400000

22120108000400000

320001000000000

19100107000400000

667811110660155971117100121

612500230003014302

612500230003014302

121130020013171192025

121130020013171192025

59459310230142770845094

59459310230142770845094

621571012000160141010

544001000106002

444001000000000

004000000000000

440001000000000

100000000106002

100000000106002

5711310110001508108

103000000000005

103000000000005

5611010110001508103

5611010110001300100

000000000208003

0176414147179204972758189639381512140

0176414147179204972758189639381512140

01173042119011377124917

0106004211901137012440

000000000070000

01130000000000517

0164711147175183961858075869279463123

018223113011022921993

038990191650000212111

000010202098201817

00000000000140740

01006050004400110

04040855143383801982142302

080400090800687003356

0190000000000000

0000000000020000

000011010070161002

0101515053200300150

070101000213010130209911

04006723117000081

191215101003090170053

19121510100309070053

19121510100309070053

19121510100309070053

0000000000010000

0000000000010000

0000000000010000

2812361010100430600049

2812361010100430600049

121002000009000

121002000009000

271035108100430510049

2110020001509005

003000000500007

24860050009070011

001000100000003

1119101000130310023

000000000004000

005000000100000

54177132153416837715398177484158499497680

29367521519167299151971718115280105168

4586750490101913703

4586750490101913703

020002000000002

020002000000002

002012312063015002011

002012312063015002011

028002001200000000

028002001200000000

0104240640000000

010040640000000

0004200000000000

0005700000000000

0005700000000000

1410202010201000

200000000101000

1210202010100000

1394124052027709115681517214160

0001800000000000

1387123872027709115681517214160

070000000000000

233163080990005202202

233163080990005202202

24896300151782164036419392512

2914102012101280287129

00300110070110110

2350103000800100

697108001130176019

005000000400000

005000000400000

16667268101441153296366311445

0000000000001800

001000001100009

298000700010092010

1540001000101100

240003000000000

200000000101002

02100210000107013201155

849208010110153029

002000000100131010

0150000003020016

0019000000230150023

0010000000200190025

4395005000903000

010000000100006

10347504001596623083

1142102000102002

110000000107002

0000000000015008

9310002000605009

0042001103490483050

11130003000200000

11150030007014006

2491005000703000

399030160001600005

399030160001600005

0017000000240251033

003000000000002

0014000000240251031

1460006000200000

1460006000200000

4161022210496733785403038

4161022210496733785403038

001000000000002

001000000000002

000000000106001

000000000106001

2751142733000203

1330011432000202

710011200000001

400020001000000

311100100000000

380006000150000

380006000150000

363866908500049014106

320102000000000

3981103000809003

21150106000600000

5490009000601000

16639550430001904103

8013010220001000000

007000000120160019

000000000202000

001000000000003

003000000106001

0030000009080015

1220001000400000

1220001000400000

1003001000600002

1003001000600002

114001000504005

114001000504005

6838042835167262109710821814929439893820177174638

172385232112595324929162722145957331863174137

1010100140400011

1010100140400011

017311120548025147195503160

017311120548025147195503160

9502821013731142700403240491

030000002000000

9472821013731122700403240491

1001089000000000

1001089000000000

320010014102501204

000010014102501104

320000000000100

891051681322681491516124622102999

902000000400004

5546116021116023102511642401960

635422023101190103046

19213506127000366055920826

031273020251020251120163

443004001100000

443004001100000

1520204000207000

1520204000207000

504934390470113261445130642

504934390470113261445130642

2023135809113420309014

930002000400000

930002000400000

6352020011025104

6352020011025104

114000000300006

114000000300006

039400002504803

009100000000000

030300002504803

1861802040002501000

1310000000300000

1731702040002201000

035001110400001

035001110400001

0012000000000000

0012000000000000

000000000008000

000000000008000

000000000008000

2882257211121231722313140375

2778236211061231552258120346

2672236211031231521258120342

000000000100004

160003000210000

1421006000170552029

1421006000170552029

26527119240177007652605482911891084

4840064512102404

4840064512102404

1160200000000000

1160200000000000

2108001100010023104

2108001100010023104

47126311600512153010

106100004215109

4612021160011000201

32000000000107900

32000000000107900

0100012400000000

0100012400000000

060001000100000

060001000100000

5401020001300000

5401020001300000

1900000000300000

1900000000300000

0090000003017004

0090000003017004

004000000000000

004000000000000

4156018302511110308

4156018302511110308

1371891413955648351284812491038

431003000001000

2320274103504375001002

110166113354561038214312471036

47900410090120016

47900410090120016

921001000300000

921001000300000

1000010001240000

1000010001240000

014001000000000

014001000000000

110000000407000

110000000407000

110000000407000

15343021271111321260028

002401011023020027

002401011023020027

15346202700119124001

15346202700119124001

24299286077212660534055

24299286077212660534055

24299286077212660534055

24299286077212660534055

516001000400108

13453020432123005200

9944203032000260481044

412101000600003

579459511597701193190521588783314

479449511597701193190421588703314

479449511597701193190421588703314

479449511597701193190421588703314

000000000000400

000000000000400

4796951159770119319000158703312

00023000500057000

000500000000000

0301900000000000

0061498083000046050

00011000000000000

01601573300215120005740430

0180036021000060000

0000291021900000061

00001000000420130

000703104923600008701440

11902473220254000058601151

040000000000040

0006510000000000

300000000000000

0904000000000000

000600000000000

0004600000000000

0004000000000010

010010900000000000

0000208211652000135000

0000000000002501

000000000000400

0000000000002101

00380000004212701

00380000004212701

0000000000001400

0000000000001400

000000000000800

000000000000800

000000000000800

000000000000800

000000000000800

101000000100000

101000000100000

101000000100000

101000000100000

101000000100000

4401955922271615668714132761699201043

4401955922271615668714132761699201043

4401955922271615668714132761699201043

4401955922271615668714132761699201043

4401955922271615668714132761699201043

4401955922271615668714132761699201043

87123332884106698657461010614478122125601661311404642

87123332884106698657461010614478122125601661311404642

87123332884106698657461010614478122125601661311404642

8685732877106696197249900647522211561616271363242

131032870471010110322000002

131032870471010110322000002

0000000110020803

0000000110020803

00000001000016012

00000001000016012

738477101996096239899527491154161631363225

181061019835662387653859510161511336924

723710125301134141540154122631

137670246222068029024044544140

137670246222068029024044544140

125000320268000143202540

0240038222800000000

0440057040060000560

090013010000000650

04900100140009720210

0070170210230000120

00002301800004502090

00005604900000000

0000000000000170

907174962393775138051392971700575278397227

907174962393775138051392971700575278397227

907174962393775138051392971700575278397227

89139356206480390701716508

89139352006480390701716508

000500000000100

634611101600015101200

269334406320390550715208

0004200000000000

0004200000000000

15651403937103380582280697568039723

15651403937103380582280697568039723

0420019242180000026804580

1523140372861361682280697300035143

0000170900000000

000000010000400

000000010000400

000000010000400

000000000000004

000000000000004

000000000000004

01300000171120109012

01300000171120109012

01300000171120109012

512451118101340413081

000000000000004

000000000000004

000000000000004

000000000000004

000000000000004

002401000060170021

002401000060170021

0021000000408006

0021000000408006

0021000000408006

0030100002090015

0000100002090015

0000100002090015

003000000000000

003000000000000

512211018101280243056

512211018101280243056

1500011100000300

1500011100000300

1500011100000300

027000000501000

027000000501000

027000000501000

4514107001230230056

4514107001230230056

4514107001230230056

38121331801190261019

38121331801190261019

38121331801190261019

38121331801190261019

151331301180261019

12421001150191014

039103000307005

3770005000100000

3770005000100000

12294575424533463291661510661011981641

6872092162511501503107226590206

571475017100213135605

571475017100213135605

571475017100213135605

571475017100213135605

630195209201133050289421330201

170741032137010921790080

13057842131010811700056

9652771128010741700056

3457103000700000

221000000209006

221000000209006

2830004000100000

2830004000100000

1012180020008000018

1012180020008000018

46012110618096040197313430121

10339206000320440044

10339206000320440044

10936140220002001101

10936140220002001101

1260005000700000

1260005000700000

3297666120630401383892076

3297666120630401383892076

343120109121112220145013720136

302831510060020550211025

302831510060020550211025

302831510060020550211025

56321020120001006001

1993556034010380151020

40130209010600004

730005000100000

833002000400003

003000000300003

003000000300003

003000000300003

830002000100000

830002000100000

830002000100000

333491215020086011610108

333491215020086011610108

920000200100000

920000200100000

814361141000310171038

1200012000000000

1017002000120110016

61219112700019061022

8813003000270280025

060003000400000

8213000000230280025

4027100000240510027

20300000080190010

0015000000100000

209100000150320017

4101500600030200018

350001000000005

120000000000000

031500500030200013

178151060013110170026

310001000000000

310001000000000

310001000000000

310001000000000

147151050013110170022

147151050013110170022

147151050013110170022

147151050013110170022

000000000000004

000000000000004

000000000000004

000000000000004

981758111101137696851064080

981758111101137696851064080

981758111101137696851064080

00000003009039104

00000003009039104

9817581111083768785673076

2060120003021100

120010002008125503

750004000100000

000000000000300

3337000000260200043

106000000701000

01000100019018000

7389004063207924414030

0000000100206108

0000000100206108

0000000100206108

0000000100206108

0000000100206108

8410314460670100731145121185

841031446067010073114551185

1352110609060161017

1352110609060161017

1352110609060161017

577117105000055112930167

577117105000055112930167

577117105000055112930167

6621640110101200111

6621640110101200111

6621640110101200111

000000000000700

000000000000700

000000000000700

000000000000700

173701915156600226645229280755147162014672472216

032400000020100016

030000000000000

030000000000000

030000000000000

030000000000000

000000000000003

000000000000003

000000000000003

000000000000003

002400000020100013

002400000020100013

002400000020100013

002400000020100013

0027000010160364090

0070000002000010

0070000002000010

0070000002000010

0070000002000010

00900000060240057

0000000001000026

0000000001000026

0000000001000026

00900000050240031

00900000050240031

00900000050240031

000000000100007

000000000100007

000000000100007

000000000100007

0011000010500407

0011000010200407

0011000010200407

0011000010200407

000000000300000

000000000300000

000000000300000

000000000000009

000000000000009

000000000000009

000000000000009

0000000002012000

0000000002012000

0000000002012000

0000000002012000

000040608280319417

0000406082000040

0000406082000040

0000406082000040

0000406082000040

0000000000009012

0000000000009012

0000000000009012

0000000000009012

0000000008031005

000000000100003

000000000100003

000000000100003

0000000004018002

0000000004018002

0000000004018002

0000000003013000

0000000003013000

0000000003013000

000000000010800

000000000010800

000000000010800

000000000010800

000000000010800

173671864156596226639228198729146154312572432093

173671864156596226639228198729146154312572432093

1812941304003250201056001503

1812941304003250201056001503

1812941304003250201056001503

2038300000350340029

2038300000350340029

2038300000350340029

04201422402394293004023030

04201422402394293004023030

04201422402394293004023030

0084000000180870045

0067000000180870045

0067000000180870045

0017000000000000

0017000000000000

224800000012070025

224800000012070025

224800000012070025

129125712537122040018673501461631254940276

03504448054101250000670

03504448054101250000670

116242202020500142141185

01100000000000410

1524220202050014210185

0008320332417000124177

0008320332417000124177

0550000000014600077

0550000000014600077

01310712331872584722000017587

01310712331872584722000017587

0541505831551290021030570

0541505831551290021030570

3153001000260210060

3153001000260210060

3153001000260210060

8113011100086017100155

8113011100086017100155

8113011100086017100155

00310000001811621035

00310000001811621035

00310000001811621035

000000000000800

000000000000800

000000000000800

00310000001811613035

00310000001811613035

00310000001811613035

000000000000300

000000000000300

000000000000300

000000000000300

000000000000300

000000000000300

16250207065764248720700764017930

16250207065764248720700764017930

16250207065764248720700764017930

16250207065764248720700764017930

0710001000000000

0710001000000000

15540207059853798720700652017090

014704772170140131010014303240

13650138728151747410600509013320

0420206100065000000530

000059045000000380

000059045000000380

000000000001120460

000000000001120460

12637133605262001010209370183

42249040346200620169270127

211130016000120311017

211130016000120311017

1490030004019009

1490030004019009

17400130008012108

0500090006012105

124004000200003

000000000000005

000000000000005

000000000000005

000000000000005

40137740186200500138260105

321251201462002104025040

321251201462002104025040

321251201462002104025040

3118000000180690034

3118000000180690034

3118000000180690034

000000000000009

000000000000009

000000000000009

1001010001000011

1001010001000011

1001010001000011

30810300070160011

30810300070160011

30810300070160011

1000000003013100

1000000003013100

1000000003013100

5811020150001800700

5811020150001800700

5811020150001800700

5811020150001800700

5811020150001800700

26243003000210403056

26135003000130143032

26135003000130143032

0112002000709009

0112002000709009

260230010006053023

2600000000000000

00230010006053023

01800000080260024

01800000080260024

01800000080260024

000000000100004

01800000070260020

11439016090381785240111

11439016090381785240111

11439016090381785240111

19160030901501021024

19160030901501021024

0000000000010000

191600308015000024

0000000100002100

05230130002317753087

007000000110160040

0000000004016007

0070000007000033

05160130001217593047

0220010001000015

000000000005000

000000000000005

000001000000005

0000000000170000

010001000203104

021401000090512018

01020100041051017

01020100041051017

01020100041051017

01020100041051017

0001000000105100

0001000000105100

0101010004000017

0101010004000017

155892251634033087721249150101569720437354988598374661363618160174004755

759864089098235105408107816813526260824209260310317588878671015

291312101718407213280

291312101718407213280

6200001003000150

620000000200000

0000001001000150

2501010840100200

2501010840100200

2161200160003211130

300000000100100

1101900000010000

0000000000000130

1110012000101000

000003000000000

1640101000110000

19190315621771041551011191691

19190315621771041551011191691

012001401070070011171580

012001401070070011171580

19703161171041481002111

19703161171041481002111

133159025759141308213231162862

15010210102000430

15010210102000430

15010210102000430

620000000100000

620000000100000

620000000100000

126152025659120308112931162432

1267201602001129311602

1267201602001129311602

080024059103080000002430

080024059103080000002430

4014314326137934131071109

4014314326137934131071109

4014314326137934131071109

7302000121300000

331121232613131101061109

00100005432001000

544031266762781264165792517111631950824531736153725521112731

54353122676252222696579229111163195082453173513742551976731

200135950252301

200135950252301

002200100000000

002200100000000

013004854026600037601160

01200457402520003320760

010020200000000

00002601200000070

00000000000440330

117455311571451488020107169060461

1651730107412133742923015

1014036856138107591770126137446

0000000004102100

0000000000102100

000000000400000

15458387712910053164147587148303223

1454437751293352103550704833223

00000016114200026900

91412067000810100

050000000000000

050000000000000

412330410812303

411220210211001

001110200601302

5829090710023211000

5829090710023211000

00000003155000000

00000003155000000

0000792208242130001

0000792208242130001

350102000300100

350102000300100

00040140600000000

00040140600000000

1961338167821441100910702

1561031066961426400210700

400307177143007002

000000094000000

398729551813031791193813

2000010101020370

000000000000101011

378729541812030771812

00000000000013800

00000000000013800

291130954518979882358681649207

000002053001000

0147046112082113

003100141201300

110007005000000

1102544530110200

410102000100100

000003000000003

000001060000000

0000000010000000

16664026017674415841541900

513010840101401380

820000000200000

39200709000511501

00000202341110000

880013000000200

0000010116000000

2990506000300500

1360003000400300

630100000200100

1200041000800310

0260118410000000140

0260118410000000140

000064041000000140

000064041000000140

144124630224436227421174420

01010001800000280

14312453022431822742117112

000000000000008

0000001000000150

6412254940535766105114425114

000005110003805

48102244334749558461391618

16235717102502101

160489214480826913022463535740303655

6663532139010843113128139502

112711432904400

0320198000000000630

93350451312151111942212292219802

020009001000000

4055501170101621

4209454420125221408226920149313681214

00000021329100000

0000000110000000

000003000000000

037029182370500210

42055441119616958175913149293591112

00000005344000000

0101080333423101000

011000230001702

0000000014000000

8170199571001301300

8170199571001301300

534481239126128622947183024185413696730

71371190170001930500

000000090000000

10000205116001119601

458438226826123022848166921480391686629

46040370391811010

1413063135531781582461685309265659

1413063135531781582461685309265659

00036430220000055130

00036430220000055130

010601550295800814001080

010601550295800814001080

0101866402801400001070

0101866202301400001070

000020500000000

0700027100304000

0700027100304000

020001021222010001

000000009000000

020001021213010001

0290092074000000381

0290092074000000381

7270227534754225019802

7270227534754225019802

462411005000513600

462411005000513600

266125581387554591089125476971352

010007000000000

337121231117081117117

12136509963254890982395547234

1428512701901035731701

0000000000102000

0000000000102000

1560102000300100

1560102000300100

146346584611640896401812650374

146346584611640896401812650374

102412620403201

102412620403201

31130508000500400

31130508000500400

432117090071000700

31100601000600300

12111108000400400

000000007000000

00100112572000000

00100112572000000

1091120991122217210514574653237

364428619501607403

73716711062116310012973949234

32402219100400100

3240226100400100

0000013000000000

1231771272741400358553979452104319246330969310

281407090005102550

80911617653923930329851132463932581548182

521300030104106

328195055335226614183110133

830220020110100

9941361137614215322011196229355566

11451059129093913219306406

15515260800412303

400000000200001

642708060002141501

35451001580211911800

18033525193313102436012

0220036639235000012802300

02200232016500001280800

000013439700000001500

540200000010100

540200000010100

540200000010100

0002573720226000016301360

0002573720226000016301360

0000000000030000

0002573720226000013301360

342224874469966852724339315173957840384752090581972

2241383324491552127867959193074941903167414

05610121616407513810029504370

05610121616407513810029504370

6276045518640980210100290081010

3760407000500400

25160113128901750904290

0540338174258901930028100720

0100110013002310360

000000000002310360

0100110013000000

00092702200008000

00092702200008000

12371105240334401800078116643712

1237110454029340180007819042312

0000700410000076140

017810490422610312000000

017810490422610312000000

143316175816038770951667247266632

0275074715407100005687202351

69400029001320310590

0000000000012302270

050060608100001420

7432111090011462601

1814010604228412300

1814010604228412300

450001000200000

450001000200000

450001000200000

1126897020693830107270520455453439616641186369929

31434121312401313

31434121312401313

0000000000022000

0000000000022000

162401928210757147339192011423111222951

162401928210757147339192011423111222951

00006443600000011130

00006443600000011130

0181910616350117303010017701951

0181910616350117303010017701951

150120100037500200

150120100037500200

933347292343221624915539541699524

71334528223522162493339541699523

220211800012200001

21711181130337767162661201870144629

000100000300000

000100000300000

1120000000400000

100000000300000

0120000000100000

000000000400000

000000000400000

100100000100100

100100000100100

0101179130337767162660091501210

0101179130337767162660091501210

0149000000000955032529

0149000000000955032529

332224285844573647172301581833

332224285844573647172301581833

0010000000021000

0010000000021000

000000000000006

000000000000006

312220275742050647291571827

3517223331845304781826

001201100302000

110002010501000

021000005000000

131202010004017200

140000000062800000

021120010421701

2031010252301700100

2031010252301700100

16411877272127450717143382326912

483208506286026302

483208506286026302

483208506286026302

0000509000000500

0000509000000500

0000509000000500

00000000001400000

00000000001400000

00000000001400000

12931140127630021600

12931140127630021600

12931140127630021600

11316211733564038312131910

11316211733564038312131910

11316211733564038312131910

00120000000000110

00120000000000110

00120000000000110

0012000000000000

0000000000000110

310000000100000

310000000100000

310000000100000

310000000100000

13937439936237987485915723588446100159800355148

684349802133184366441015247125571119693278116

27220705095629201300

000100000200600

2722060509562720700

002110100100000

002110100100000

268131138046343172362400

268131138046343172362400

11020001531200100

110200000200100

00000001531000000

10100041385280004501

10100041385280004501

000100009000000

000100009000000

54501028622097250610200

0000000127000000

545010286200250610150

00000001947000000

0000000023000050

18110602000320100

18110602000320100

000000005000000

000000005000000

4526080301181521200

331908010001021200

520000000200000

3300000118200000

420002000100000

6161236190202068167833538017555

212302310101410

0000070200111360390

0000001063000000

5659301881020260462291613555

301321000004000

391611492333421120122511215

1011101156448000071

1000600022111100

00011001240000140

13901117000400901

0000000114382001000

00000203385000000

000600200202100

2470504000511000

000000038159000003

82000300001088120

8200030000100120

0000000000008800

33717431083551404952850044

33717431083551404952850044

2516173288912382733113010

0001100304012001

251617317891208233191309

1536629242372482835253248755501

011002067135000000

010008110000000

0000000000450000

153642824227247215390323755501

1796971848162253964516913179

1796971848162253964516913179

1796971848162253964516913179

060000000000000

060000000000000

060000000000000

3615412742282571847301714

720002092100200

000001092000000

720001000100200

165411735281651337261714

1514117628161103725014

000000004000000

1000000000000170

0400029000300100

1380105000410200

1380105000410200

320000000200000

320000000200000

320000000200000

11010002332000100

11010002332000100

11010002332000100

870100000500100

130000000200000

130000000200000

740100000300100

740100000300100

482294811762341624701006115192462439

10763045038014130285181301

1250303000300300

540100000010001

0000050071000000

310101000300000

875001701300021351000

03023016014591113000

402206172068125230

402206172068125230

0000000112000000

0000000112000000

113211821139320510506

16011541117196310101

071002802289200405

0010000035000000

100003047500000200

0000000070000200

10000304743000000

1205502002000129321100

421407070001320500

29130304000800300

14100301000401100

35180708001411200

19813764347815821345442922602

19813663947815821237042922601

0104000184000001

00000004133000000

00000004133000000

15402061150300200

920105000200100

6201011150100100

000000008000000

000000008000000

000000008000000

15022263441738164437143458707

201116031571457027500

201116031571457027500

201116031571457027500

0000000279000000

0000000279000000

0000000279000000

1301114181415712527341207

1301114181415712527341207

1301114181415712527341207

0005326022000030000

0005326022000030000

0005326022000030000

0000000931000000

0000000931000000

0000000931000000

7923119092392209086548680847855634825843321924528141200253443682

000008000000000

000008000000000

000008000000000

000008000000000

2684106770012529749226214621160773947968751249

610300202376197941836314328123419346

1490009000000000

1490009000000000

1880007000200000

1880007000200000

72210041005000180

72210041005000180

9933226132042301213606

8027050250002700300

211000032112105

650006000100000

2021111010000000

90000000010013201

26213905074211580131032

990104100200005

25313004070111560131027

111785164159112331612178951233

111785164159112331612178951233

1136821905501037002045

1136821905501037002045

86473111000210580024

86473111000210580024

0000000000030006

0000000000030006

207476749888235521321962684677066673456903

0000000000000013

0000000000000013

0007000000100320

000700000010000

0000000000000320

100000000000009

100000000000009

207376749881235521321962684676966673424881

282402018001503200

007100000000000

000000110602300

00831000005604510140

4547001021701200

26111322874117016409

003300001054013400161

0000012011012000

200100000000000

1703667183614692113278048152211

1102327790150211370038530536

33123110606120773410

48182350172211921310

0018000000000000

000000010000109

75177151151017311823618009

32106187173005323466606

000800000000000

000800000000000

000800000000000

000800000000000

109210202000028000

109210202000028000

0090000190008000

009000000008000

0000000190000000

1001020000020000

1001020000020000

0002000010000000

0002000010000000

1931118716643114023079101126621337483126425632751160158

40735664248771693611208397155301122109837

000000000000800

000000000000800

0000000033010000

0000000033010000

11997621814426943512294349634

11997621814426943512294349634

2081931502601616436296121431241

1319813713925130741600

000000019000000

0000001000000240

0000100157350000000

7487013048631257591101

3800076000901400

00007230504000046112680

00007230504000046112680

020000210130200

020000210130200

8057080630112636801

5235060150001614301

2822020480111022500

0719235701023001

0719235701023001

152483110218363123864002454940328123414430651062121

1063153262152140131512714312

1063153262152140131512714312

24566252485894062965736219285058

24566252485894062965736219285058

1381313280267020535920144

274615023602027135142

1118522303100026461502

2826070110001331501

100000000300000

2726070110001031501

61724319932102616140967178638522240125

5981981983270316140667153638472232123

19450103230302505802

6303017010400300

6303017010400300

0130022101220900031790

0130022101220900031790

2411510551651134061763581

2411510551651134061763581

128120135187781059084514179343

72000015400015013270

121100135186241059069513149073

110281610122901032715

110281610122901032715

373110929032591585911381011

373110929032591585911381011

000204210002000

000204210002000

5023140103900420401

5023140103900420401

2380603000220300

2380603000220300

0000188010000000000

0000188010000000000

000080034000000220

000080034000000220

600000000100000

600000000100000

600000000100000

600000000100000

7573262449419147102108101713322926815460

75663241714441581159129992499360

115102800312101

115102800312101

0410017000330000

0410017000330000

755581817042578115969590489359

41141314263450913404

75147141691115181146487449355

12630323187387278581223019610

12630323187387278581223019610

1261032318728726858121901610

020001000000100

0000000100401700

0001020115178900

0001020115178900

0001020115178900

0000000015128900

000102010005000

3651482987101031221333293630531463

3651482987101031221333293630531463

009000000000000

009000000000000

008000000100000

008000000100000

29813924171087122132799245851417

83211130014991280072

8516100200099020100137

750005000000000

000001010004000

101110011202102

760000000300000

0189000090002000

300000000100009

1081003000701100

17170020360002100300

00000000010032000

000000000300000

006000000000005

0000800008000030

002500001041011200102

000000009000000

000000000300000

0010000002035000

13214006000200006

922109141111014

201300011000500000

000100001150320019

0000010002000013

1000000000000018

4070109000300000

000001060208000

00000000000444600

00000000000444600

14312003000111442018

14312003000111442018

536280013000410840028

44400011000500000

9223002000210540016

005000000150300012

30125761842226009014760

1321613019114100200

1321613019114100200

1321613019114100200

000000106000000

000000106000000

000000106000000

10241512385243600060

10241512385243600060

10241512385243600060

78000301182372014500

78000301182372014500

78000301182372014500

779367559173011998116211010831

779367559173011998116211010831

779367559173011998116211010831

775367558172911993115209110601

4011050011900230

1369877761818083461379131682361515021516791718749438381744

6669106827382013

6669106827382013

2256105726362011

441300110102002

711683155314581010321224413200

25150054000100200

2500048000100200

000005000000000

0015001000000000

68162055314471031912441000

68162055314471031912441000

1110003020001200

1110003020001200

0015000000000000

0015000000000000

14579281601485233521012319340440640139501581702328170

1397924155148463346992201334440436139501481701321168

00019000100801501

111883220690863923821633470903

2890658178413255815118

01340000000000000

1015060669825121087581483496710512315894

0000000220000000

1588940126611086629101182317021

16021982314362234132955138

02570849199342135332539229386931180

0380101020077090200

02690139600121018500430

4015020002005003

000101000020600

645662118624010172

645662862408172

00000011000002000

30131631265315221083

30131631265315221083

100014110000102

291316285431522981

11166064192590124261782519824181512583547575734821558

01005224100037100156

01005224100037100156

1320131400010266

1320131400010266

1057105121330914882991510

83674381141262293125

020205083114121

2011145112112514

5120911111033136021016

0000000000006001

30106083030100313

211031380031202702

312267230524208169134718844312402892150222188

102000040000011

197518625017093103270322232891107464562

011236442913909612615755

0000000001101000

0009800000000000

11421519170347229435517822101950020

721821111898010414316

721821111898010414316

49223421497586272339805352078093219249173

414176202461919014648351977992189949156

0006200100000000

0000562031900001000

001150510000000

000157040000000001

785803104201560110293016

216516782021811213959

216516782021811213959

24571106146340511828337227939472109102273853

0001369382123810000000

0205001210001000

2456910689231173337027939472108102273853

11451247811042319

621103521132211

524021260910108

42145354211912732

42145354211912732

81913333190121160

6120130319021155

2071203000010005

001000210000000

001000210000000

001000210000000

1560731301500922000

056072000140000000

056072000140000000

0000000000901800

0000000000901800

100113010002200

100113010002200

120602827258204013001076

120602827258204013001076

120602827258204013001076

0060000000000730

00005006200000000

01900540450000126002

100007000200000

01001780151200000340

000000000204004

0571144142172005001

0571144142172005001

007000000000000

007000000000000

00010312172000001

00010312172000001

05001411300005000

05001411300005000

0000000000027000

0000000000027000

0000000000027000

0000000000027000

0000000000027000

008000000000000

008000000000000

008000000000000

008000000000000

008000000000000

620103000100000

620103000100000

620103000100000

620103000100000

620103000100000

624197671733416191274412771158321561845926941896

014000000100010800

014000000100010800

00000000000010800

00000000000010800

0140000001000000

0140000001000000

624183671733416191274412771157321561845916141896

900001010000100

900001010000100

900001010000100

201878015515286095428920641052941317800

0000000000001100

0000000000001100

0000000240000000

0000000240000000

000010030000100

000010030000100

201903701341900310000

2090303100310000

01000701041800000000

018590152826575352622061105194117800

0252001663810962117007805940

010070010309311800807011540

019006906000015000590

08400250151009400110

0000100000011000

01790016207970000505200

00003102400000090

0167011434022111000001520

01510382351913307910002810

1222906715742632261790986951201456536514224096

121046210881183599227116145400241130

12104621088933529227026145400241130

0000250709000000

012800101907042269002003180

012800101907042269002003180

115854861428199888171142534511819676

10352104601404002

155014970340013902519211112192

000482775155520320014907480

000115549220000702

0000674393400000110

0000620353300000110

000054410000000

21150414010810500

21150414010810500

21150414010810500

0027001000240191052

0027001000240191052

0027001000240191052

0027001000240191052

0027001000240191052

151531293984847212221341075177

146180040001404324041

1331100000010221902

133110000001018002

133110000001018002

133110000001018002

0000000000041900

000000000004000

000000000004000

0000000000001900

0000000000001900

02700200060170021

02700200060170021

02700200060170021

02700200060170021

1100020007045018

110001000502008

110001000502008

110001000502008

000000000200500

000000000200500

000000000200500

0000010000020010

0000010000020010

0000010000020010

11213000040301107

1020000000201000

1020000000201000

1020000000201000

1020000000201000

1013000000100007

005000000000000

005000000000000

005000000000000

108000000100007

108000000100007

108000000100007

000000040000100

000000040000100

000000040000100

000000040000100

451829388254223623477547

1120503000190517011

1120503000190517011

1120503000190517011

1120503000190517011

481238942222151456

481238942222151456

0000050100201401

000005000000201

0000000100201200

411004000200003

001001000200003

410003000000000

07023804120015052

07023804120015052

306001000607406

306001000607006

306001000607006

306001000607006

000000000000400

000000000000400

000000000000400

1240100800050142016

1240100800050142016

124010080001002700

124010080001002700

0000000004010016

0000000004010016

0000000000001500

0000000000001500

2552004000406008

2450004000300000

2450004000300000

2450004000300000

000000000103000

000000000103000

000000000103000

102000000003008

102000000003008

102000000003008

8127691019010690565082

8127691019010690565082

8127691019010690565082

8127691019010690565082

8127691019010690565082

383827717108112856617185011016

383827717108112856617185011016

383827717108112856617185011016

383827717108112856617185011016

383827717108112856617185011016

35251709000300800

313260179911285661418503016

36223007000161022560207

36223007000161022560207

36223007000161022560207

36223007000161022560207

36223007000161022560207

2511300500074013050107

111100020008709510100

0000000000109600

0000000000109600

0000000000109600

0000000000109600

0000000000109600

0000000000109600

4049166075000550571012

4049166075000550571012

4049166075000550571012

44110009000600002

44110009000600002

44110009000600002

8613010130001000000

8613010130001000000

8613010130001000000

27467050530003103100

27467050530003103100

27467050530003103100

00600000080540010

00600000080540010

00600000080540010

000000000000300

000000000000300

000000000000300

000000000000300

000000000000300

000000000000300

0000204240001008

0000204240001008

0000204240001008

0000204240001008

0000204240001008

0000204240001008

0000204240001008

411205216278080012703

0040107790100000

0040107790100000

0040107790100000

0000000100000000

0000000100000000

0000000100000000

0040107690100000

0040107690100000

0040107690100000

417001000000002

417001000000002

417001000000002

417001000000002

107001000000001

107001000000001

310000000000001

310000000000001

0010317195050012701

0010317195050012701

0010317195050012701

0010317195050012701

0010317195050012701

0010317195050012701

000010240200000

000010240200000

000010240200000

000010240200000

000010240200000

000010240200000
